# Evaluating Acupuncture for Chemotherapy-Induced Peripheral Neuropathic Pain: A Narrative Review of Clinical Evidence and Mechanistic Perspectives

**DOI:** 10.3390/healthcare14183077

**Published:** 2026-09-18

**Authors:** Paula Sousa, Madalena Deus, Mariana Pedrosa, Jorge Magalhães Rodrigues, Fabricio Pereira, Maria Begoña Criado, Jorge Pereira Machado, António Moreira

**Affiliations:** 1 ICBAS, School of Medicine and Biomedical Sciences, University of Porto, 4050-313 Porto, Portugal; 2 CBSin, Centre of Biosciences in Integrative Health, 4250-105 Porto, Portugal; 3 MIPE, Université de Nîmes, 30000 Nimes, France; 4 Associate Laboratory i4HB—Institute for Health and Bioeconomy, University Institute of Health Sciences, 4585-116 Gandra, Portugal; 5 UCIBIO—Applied Molecular Biosciences Unit, Toxicologic Pathology Research Laboratory, University Institute of Health Sciences (1H-TOXRUN, IUCS-CESPU), 4585-116 Gandra, Portugal; 6 Sport Sciences School of Rio Maior, Santarém Polytechnic University, 2040-413 Rio Maior, Portugal; 7 Research Center in Sports Sciences, Health Sciences and Human Development (CIDESD), Santarém Polytechnic University, 2001-904 Santarém, Portugal

**Keywords:** acupuncture, chemotherapy-induced peripheral neuropathy, neuropathic pain, neurotoxic damage

## Abstract

**Highlights:**

**What are the main findings?**

- Current evidence suggests that acupuncture may reduce pain and sensory symptoms in some patients with painful chemotherapy-induced peripheral neuropathy; however, confidence in these findings is limited by small sample sizes, heterogeneous protocols, variable control conditions, and short follow-up periods.

**What are the implications of the main findings?**

- Acupuncture may be considered a complementary component of individualized, multimodal care, while its precise clinical role and the patient groups most likely to benefit remain to be established in rigorously designed trials.

**Abstract:**

Background/Objectives: Despite the high prevalence of chemotherapy-induced peripheral neuropathic pain (CIPNp), its management remains challenging, and reliable preventive strategies are still lacking. Current treatment is largely symptomatic and often provides only modest benefits, as available pharmacological therapies have shown limited and inconsistent efficacy. In this context, acupuncture has been investigated as a non-pharmacological intervention, but the certainty and clinical applicability of the available evidence remain unclear. This critical narrative review examines the clinical evidence, mechanistic plausibility, and current research gaps concerning acupuncture for CIPNp. Methods: A non-systematic narrative review was conducted with preference given to peer-reviewed articles published in English, including clinical trials, systematic reviews, meta-analyses, clinical guidelines, mechanistic studies, and narrative reviews considered relevant to the objectives. Publications were not selected according to a predefined systematic protocol, and no formal quality assessment, risk-of-bias analysis, or meta-analysis was performed. The extracted information was organized thematically rather than quantitatively. Results: Clinical trials and evidence syntheses suggest that acupuncture may be associated with reductions in pain and sensory symptoms in some patients with CIPN. However, confidence in these findings is limited by small samples sizes, heterogeneous acupuncture protocols, variable comparators and outcome measures, and limited long-term follow-up. Much of the proposed mechanistic rationale derives from animal models or human studies in non-CIPN pain conditions. Conclusions: Acupuncture may be considered a monitored adjunctive option for selected patients with persistent CIPNp. More rigorous and adequately powered trials are required to determine efficacy, optimal treatment protocols, durability of benefit, and potential treatment-response modifiers.

## 1. Introduction

Chemotherapy-induced peripheral neuropathy (CIPN) is a frequent and often dose-limiting complication of modern anticancer therapies, particularly those involving platinum compounds, taxanes, vinca alkaloids, proteasome inhibitors, epothilones, and immunomodulatory agents [1,2,3]. Although advances in oncology have substantially improved survival rates, they have also led to a growing population of cancer survivors living with long-term treatment-related toxicities. Among these, CIPN represents one of the most burdensome conditions, characterized by persistent sensory dysfunction, neuropathic pain, motor impairment, and, in some cases, autonomic involvement, with profound consequences for daily functioning and quality of life [2,4].

Epidemiological studies consistently demonstrate that CIPN affects a substantial proportion of patients during and after chemotherapy, with prevalence estimates varying widely according to the neurotoxic agent, cumulative dose, and assessment methodology [1,5]. Importantly, CIPN frequently persists beyond the completion of treatment and may even worsen after chemotherapy cessation, a phenomenon known as “coasting” [1,2]. This persistence transforms CIPN from an acute adverse effect into a chronic neurological condition, commonly accompanied by sleep disturbances, anxiety, depressive symptoms, reduced physical activity, and loss of functional independence [4].

The principal chemotherapeutic agents associated with CIPN in different types of cancer are summarized in Table 1. The proposed mechanisms of action and distinct clinical phenotypes of chemotherapy-induced peripheral neuropathy associated with specific chemotherapeutic agents are summarized in Table 2.

Beyond its impact on individual patients, CIPN has major implications for oncological care. Neuropathic symptoms are among the most frequent causes of chemotherapy dose reduction, treatment delay, or premature discontinuation, directly affecting treatment efficacy and survival outcomes [1,3]. Despite this clinical relevance, effective prevention and management strategies remain limited. Pharmacological treatments, largely extrapolated from other neuropathic pain syndromes, have shown modest and inconsistent benefits in CIPN, reflecting fundamental differences in underlying pathophysiology and highlighting a significant unmet clinical need [2,6].

In response to these limitations, increasing attention has been directed toward non-pharmacological and integrative approaches for CIPN, including exercise-based rehabilitation, cryotherapy, neuromodulation, and acupuncture [7,8]. Among these interventions, acupuncture has gained particular prominence due to its favorable safety profile and evidence suggesting possible benefit in a range of chronic pain conditions and peripheral neuropathies [9,10]. Within oncology supportive care, acupuncture has also been associated with improvements in cancer-related symptoms such as pain, fatigue, nausea, anxiety, depression, and sleep disturbances [2].

Importantly, recent high-level evidence has strengthened the rationale for acupuncture specifically in the context of CIPN. The systematic review and meta-analysis by Zhao et al. [11] reported significant improvements in neuropathic symptoms in patients with chemotherapy-induced peripheral neuropathy, including pain severity, sensory dysfunction, and overall neurological function, while maintaining a high margin of safety. These findings provide quantitative support for acupuncture as a potentially beneficial adjunctive option, while simultaneously underscoring the methodological heterogeneity of existing studies. Nevertheless, despite growing evidence, the literature on acupuncture for CIPN remains fragmented. Considerable variability exists with respect to acupuncture modality, point selection, treatment frequency and duration, outcome measures, and control conditions, limiting direct comparison across studies [8,9,10]. Moreover, many reviews emphasize clinical outcomes without sufficiently integrating contemporary understanding of CIPN pathophysiology or the neurobiological mechanisms through which acupuncture may exert its effects. This gap contributes to ongoing skepticism and hampers translation into routine clinical practice [2].

Furthermore, CIPN is increasingly recognized as a heterogeneous condition arising from a complex interaction of predisposing, precipitating, and perpetuating factors, including metabolic comorbidities, treatment-related neurotoxicity, central sensitization, and psychosocial contributors [1,3]. However, few reviews explicitly address how integrative interventions such as acupuncture may interact with these mechanisms or which patient subgroups are most likely to benefit.

Against this background, a comprehensive and integrative synthesis of the literature is warranted. The present review aims to critically examine the epidemiology, clinical features, diagnostic challenges, and therapeutic approaches to chemotherapy-induced peripheral neuropathic pain, with particular emphasis on acupuncture as a non-pharmacological intervention. By integrating clinical evidence with emerging insights from neurobiology and neuroimaging, this review seeks to clarify current knowledge gaps, contextualize acupuncture within a multimodal management framework, and identify priorities for future research and clinical implementation.

## 2. Materials and Methods

### 2.1. Rationale for a Narrative Review Approach

This work was designed as a non-systematic narrative review to provide an integrative synthesis of the current literature on CIPNp, with particular emphasis on acupuncture as a non-pharmacological therapeutic intervention. A narrative review approach was chosen rather than a systematic review for several reasons. The available literature on acupuncture for CIPNp is marked by substantial heterogeneity across multiple dimensions: chemotherapeutic agents, CIPN phenotypes, acupuncture modalities, treatment protocols, outcome measures, and control conditions. This heterogeneity, while limiting the feasibility of meta-analysis, reflects genuine clinical complexity and variability in real-world practice. A narrative review approach permits systematic examination of this heterogeneity, identification of emergent themes, and integration of diverse evidence types—including mechanistic studies, clinical guidelines, and patient-centered evidence—that would be difficult to synthesize within the constraints of a formal systematic review protocol. Nevertheless, we acknowledge important limitations of the narrative approach, including potential selection bias, subjective interpretation, and lack of formal quality assessment. To address these limitations, we have endeavored to make the literature-identification process as transparent as possible, present findings critically with explicit discussion of methodological limitations, and to distinguish clearly between preliminary evidence and established clinical efficacy.

### 2.2. Literature Search Strategy

Relevant literature was identified through targeted searches of four major electronic databases: PubMed/MEDLINE, Scopus, Web of Science, and Google Scholar. These platforms were selected to ensure comprehensive coverage of peer-reviewed biomedical literature. Searches were conducted using combinations of controlled vocabulary (Medical Subject Headings for PubMed) and free-text terms. The search strategy encompassed three conceptual dimensions:

Peripheral neuropathy domain: chemotherapy-induced peripheral neuropath, CIPN, peripheral neuropath, neuropathic pain, neurotoxic, chemo-induced neuropath, cancer-related neuropathy.

Intervention domain: acupuncture, electroacupuncture, electro-acupuncture, needle acupuncture, traditional acupuncture.

Study type filters: clinical trial, randomized controlled trial, RCT, systematic review, meta-analysis, evidence synthesis, guideline, mechanistic studies, neurobiological mechanism.

These terms were combined using Boolean operators to construct comprehensive search queries adapted for each database’s search syntax. In addition to database searches, reference lists from key articles, systematic reviews, and meta-analyses were manually screened to identify additional relevant publications. Literature searches were conducted through October 2025. No review protocol was preregistered, consistent with the non-systematic nature of the narrative review.

### 2.3. Eligibility Criteria

Inclusion Criteria

Publications were included if they met the following criteria: (i) Study type: peer-reviewed articles including clinical trials (randomized controlled trials, non-randomized trials, observational studies), systematic reviews, meta-analyses, clinical practice guidelines, mechanistic studies (animal models, in vitro studies, neuroimaging studies), narrative reviews, and editorials addressing treatment rationale; (ii) Population: adult patients (≥18 years) with CIPN or CIPNp, or animal models/mechanistic studies directly relevant to understanding CIPN pathophysiology or acupuncture’s neurobiological mechanisms; (iii) Intervention: acupuncture, electroacupuncture, or related traditional acupuncture-based modalities; (iv) Outcomes: primary outcomes related to pain intensity, sensory symptoms, or neuropathic pain; secondary outcomes including functional impairment, quality of life, adverse effects, or mechanistic endpoints; (v) Language: publications available in English; (vi) Publication date: no restriction; all years of publication were considered.

Exclusion Criteria

Publications were excluded if they: (i) addressed other neuropathies not specifically related to chemotherapy exposure; (ii) examined acupuncture for cancer-related symptoms other than CIPN/CIPNp (e.g., cancer pain, chemotherapy-related nausea, fatigue) without specific CIPNp outcome data; (iii) were opinion pieces, editorials, or commentaries without substantive evidence review; (iv) lacked sufficient methodological detail or outcome data for interpretation; (v) were not available in English or as full-text articles.

### 2.4. Approach to Quality Assessment and Critical Appraisal

A formal systematic quality assessment tool was not applied uniformly across all included studies. This reflects both the diversity of study designs included (ranging from animal mechanistic studies to clinical trials to narrative reviews) and the exploratory, thematic nature of the narrative review approach. Consequently, the findings should be interpreted as a critical thematic synthesis rather than as a comprehensive or quantitatively pooled estimate of treatment efficacy.

Organization and Synthesis

Rather than presenting results according to formal meta-analytic synthesis or hierarchical GRADE levels of evidence, findings were organized thematically by topic (e.g., clinical efficacy, mechanistic pathways, specific drug-related phenotypes) to facilitate integration of heterogeneous evidence and highlight areas of agreement and disagreement across studies.

## 3. Results

### 3.1. Etiology of Chemotherapy-Induced Peripheral Neuropathic Pain

CIPNp arises from the neurotoxic effects of antineoplastic agents on the peripheral nervous system and reflects a complex interaction between patient-related vulnerability factors, drug-specific mechanisms of neurotoxicity, and biological processes that sustain nerve dysfunction over time [1,2,3]. Rather than representing a uniform clinical entity, CIPNp encompasses a spectrum of phenotypes whose expression depends on the chemotherapeutic agent used, cumulative exposure, and individual susceptibility.

#### 3.1.1. Predisposing Factors

Several patient-related factors predispose individuals to the development of CIPNp. Advanced age is consistently associated with increased risk, likely reflecting reduced neuronal reserve, impaired axonal regeneration, and greater mitochondrial vulnerability [2,12]. Metabolic comorbidities, particularly diabetes mellitus and thyroid dysfunction, contribute to pre-existing peripheral nerve compromise and significantly increase the likelihood and severity of CIPNp [3,6]. Nutritional deficiencies, including avitaminosis affecting B-complex vitamins, as well as pre-existing neuropathies related to neoplasia or previous neurotoxic treatments, further lower the threshold for chemotherapy-induced nerve injury [1,4]. Concomitant use of potentially neurotoxic medications, such as metronidazole, misonidazole, sulfasalazine, or phenytoin, may also potentiate peripheral neurotoxicity [6].

Genetic susceptibility has increasingly been recognized as an additional predisposing factor, with polymorphisms affecting drug metabolism, mitochondrial function, inflammatory signaling pathways, and ion channel regulation influencing individual vulnerability to CIPNp [2]. These predisposing factors help explain why some patients develop severe neuropathic pain after relatively modest chemotherapy exposure, whereas others tolerate higher cumulative doses with minimal symptoms.

#### 3.1.2. Precipitating Factors: Drug-Specific Neurotoxicity

The precipitating factors underlying CIPNp are primarily related to the direct neurotoxic effects of specific chemotherapeutic agents. Different drug classes induce peripheral nerve damage through distinct, although partially overlapping, mechanisms [3,5].

Platinum compounds, particularly oxaliplatin, preferentially accumulate in the dorsal root ganglia and induce sensory neuron toxicity through mitochondrial dysfunction, oxidative stress, and dysregulation of sodium and calcium channels. Clinically, this results in a predominantly sensory neuropathy characterized by intense pain, cold-induced allodynia, dysesthesia, and muscle cramps related to peripheral nerve hyperexcitability [2,12].

Taxanes, such as paclitaxel and docetaxel, disrupt microtubule dynamics and axonal transport, leading to distal axonal degeneration and pronounced neuroinflammatory responses. In contrast to platinum-induced neuropathy, taxane-related CIPNp is strongly associated with neuroinflammation and peripheral nerve sensitization, contributing to persistent neuropathic pain [6,12]. Vinca alkaloids similarly interfere with microtubule assembly, while proteasome inhibitors and immunomodulatory agents exert additional neurotoxic effects through mitochondrial impairment and inflammatory pathway activation [2].

Treatment-related variables—including cumulative dose, dose intensity, infusion schedule, and combination regimens—play a critical role in modulating the risk and severity of CIPNp. Combination therapies involving multiple neurotoxic agents are associated with a higher likelihood of developing clinically significant neuropathy [1,3].

#### 3.1.3. Persistence and Perpetuating Mechanisms

Importantly, the clinical course of CIPNp cannot be fully explained by the initial peripheral nerve injury. A substantial proportion of patients experience persistent or even worsening symptoms after cessation of chemotherapy, a phenomenon known as coasting [1,2]. This observation indicates the involvement of perpetuating mechanisms that maintain neuropathic pain beyond the period of active neurotoxic exposure. The prevalence of CIPNp after completion of chemotherapy in different studies is shown in Table 3.

Perpetuating factors include sustained neuroinflammation, maladaptive plasticity within the peripheral and central nervous systems, and dysregulation of pain modulatory networks. Persistent activation of immune and glial cells, ongoing release of pro-inflammatory cytokines, and altered expression of ion channels and neurotrophic factors contribute to prolonged peripheral sensitization [2,12]. Concurrently, central sensitization processes—manifested by altered spinal nociceptive processing and dysfunction of supraspinal networks involved in sensory integration, affective regulation, and executive control—play a crucial role in the chronicity and amplification of CIPNp symptoms [3]. Psychological and behavioral factors further reinforce symptom persistence. Sleep disturbance, anxiety, depressive symptoms, fear-avoidance behaviors, and reduced physical activity are frequently observed in patients with chronic CIPNp and are increasingly recognized as active contributors to pain maintenance rather than secondary consequences [1,4].

#### 3.1.4. Etiological Implications for Integrative Interventions

From an etiological standpoint, the multifactorial and self-perpetuating nature of CIPNp provides a strong rationale for integrative therapeutic approaches. The recent systematic review and meta-analysis by Zhao et al. [11], showing significant improvements in pain and sensory dysfunction with acupuncture, aligns with this etiological framework. Rather than targeting the initial neurotoxic insult alone, acupuncture may modulate key perpetuating mechanisms—including neuroinflammation, peripheral and central sensitization, and affective components of pain—thereby addressing domains insufficiently targeted by pharmacological therapies.

In summary, CIPNp results from the interaction of predisposing patient factors, drug-specific neurotoxic mechanisms, and perpetuating biological and psychosocial processes. Recognizing this etiological complexity is essential for understanding disease heterogeneity and for guiding mechanism-informed, multimodal management strategies.

### 3.2. Diagnosis and Assessment of Chemotherapy-Induced Peripheral Neuropathic Pain

The diagnosis and assessment of CIPNp remain clinically challenging due to the heterogeneity of symptoms, the absence of validated objective biomarkers, and the complex interaction between peripheral nerve injury and central nervous system mechanisms [1,2,3]. At present, CIPNp is primarily a clinical diagnosis, based on the integration of patient-reported symptoms, neurological examination, and complementary assessment tools. Clinically, CIPNp typically presents as a symmetrical, length-dependent sensory neuropathy with a “glove-and-stocking” distribution. Common symptoms include paresthesia, numbness, dysesthesia, burning pain, electric shock–like sensations, and, in some cases, motor weakness or autonomic dysfunction. Symptom severity may fluctuate during treatment and frequently persists after chemotherapy completion, complicating longitudinal assessment and follow-up [1,2].

Neurological examination remains a cornerstone of assessment and includes evaluation of light touch, pinprick, vibration, temperature sensation, deep tendon reflexes, muscle strength, balance, and gait. However, several studies have demonstrated a poor correlation between objective neurological findings and patient-reported symptom burden, particularly in chronic CIPNp, highlighting the limitations of examination alone in capturing the lived experience of neuropathic pain [3].

Clinician-based grading systems, most notably the National Cancer Institute Common Terminology Criteria for Adverse Events (NCI-CTCAE), are widely used in oncology practice to document neurotoxicity. While these scales facilitate standardized reporting, they are relatively insensitive to subtle sensory changes, functional impairment, and pain severity, and often underestimate the true clinical impact of CIPNp [2]. As a result, reliance on clinician-graded toxicity scales alone may lead to underdiagnosis and delayed intervention.

To address these limitations, patient-reported outcome measures (PROMs) have gained increasing importance in both clinical practice and research. Instruments such as the EORTC QLQ-CIPN20 and the Functional Assessment of Cancer Therapy/Gynecologic Oncology Group–Neurotoxicity (FACT/GOG-NTx) questionnaire allow for systematic assessment of sensory, motor, and functional symptoms from the patient’s perspective and have demonstrated greater sensitivity in detecting CIPN-related impairment compared with clinician-based scales [1,2]. Neuropathic pain-specific tools, including the DN4 questionnaire, may further support the identification of neuropathic pain features and symptom characterization.

Neurophysiological investigations can provide objective information regarding peripheral nerve function and are particularly useful in selected cases. Nerve conduction studies (NCS) are considered the gold standard for evaluating large-fiber neuropathy and may reveal axonal degeneration or conduction abnormalities associated with CIPNp [3]. However, NCS have limited sensitivity for small-fiber dysfunction, which plays an important role in neuropathic pain; therefore, normal results do not exclude clinically significant CIPNp.

Additional diagnostic modalities, such as quantitative sensory testing (QST) and skin biopsy for intraepidermal nerve fiber density, have been employed mainly in research settings. QST allows for psychophysical assessment of thermal and mechanical thresholds and may capture both large- and small-fiber dysfunction, while skin biopsy has demonstrated reduced nerve fiber density in patients exposed to neurotoxic chemotherapy agents [2]. Nevertheless, limited availability, lack of standardization, and uncertain clinical thresholds currently restrict their routine use.

Importantly, growing evidence suggests that CIPNp involves not only peripheral nerve injury but also maladaptive central nervous system changes, including altered spinal processing and dysfunction of supraspinal pain-modulating networks [2]. These central mechanisms may explain the frequent dissociation between peripheral nerve damage and symptom severity, as well as the persistence of pain after chemotherapy cessation. In this context, functional neuroimaging techniques such as functional magnetic resonance imaging (fMRI) have been explored as research tools to investigate altered connectivity within pain-related brain networks. Although promising, neuroimaging remains investigational and is not currently recommended for routine clinical diagnosis of CIPNp. The heterogeneity of CIPNp phenotypes further complicates diagnostic assessment. As highlighted in Table 2, distinct clinical patterns are associated with different chemotherapeutic agents, reflecting underlying differences in neurotoxic mechanisms. Recognition of these phenotypes is clinically relevant, as it may inform prognostic expectations and guide individualized therapeutic strategies.

Notably, in acupuncture trials reported in Zhao et al. [11], diagnosis of CIPNp was predominantly based on clinical criteria combined with patient-reported outcomes rather than objective biomarkers. This underscores the current reality that symptom-based assessment remains central to both clinical management and interventional research in CIPNp. Overall, accurate diagnosis and assessment of CIPNp require a multimodal and longitudinal approach integrating clinical examination, patient-reported measures, and selected objective tests when indicated. Improved phenotypic characterization and the development of reliable biomarkers remain critical unmet needs and represent important priorities for future research.

The most commonly used clinical, patient-reported, and neurophysiological tools for the assessment of CIPNp are summarized in Table 4.

### 3.3. Therapeutic Approaches to Chemotherapy-Induced Peripheral Neuropathic Pain

#### 3.3.1. Pharmacological Treatments: Limited Efficacy and Unmet Needs

The pharmacological management of CIPNp remains limited and largely unsatisfactory. Despite the high prevalence and clinical impact of CIPNp, no pharmacological agent has demonstrated consistent efficacy for prevention, and available treatments offer, at best, modest symptomatic relief once neuropathy is established [1,2,6]. Most pharmacological strategies currently used in CIPNp are extrapolated from treatments for other neuropathic pain conditions, despite important differences in underlying pathophysiology. Antidepressants, anticonvulsants, opioids, and topical agents have all been investigated, with generally inconsistent and often disappointing results [2,13]. Among these agents, duloxetine is the only drug supported by moderate-quality evidence for the treatment of CIPNp. Randomized controlled trials have shown a modest reduction in pain severity compared with placebo, particularly in patients with oxaliplatin-induced neuropathy [6,14]. However, the magnitude of benefit is limited, responses are heterogeneous, and efficacy appears to vary across chemotherapeutic agents. In addition, adverse effects such as nausea, fatigue, and somnolence may restrict tolerability in a subset of patients. Other commonly prescribed medications, including tricyclic antidepressants (e.g., amitriptyline), serotonin–noradrenaline reuptake inhibitors other than duloxetine, and gabapentinoids (gabapentin and pregabalin), have failed to demonstrate significant or sustained benefit in CIPNp in randomized trials [2,6,13]. These negative findings are particularly relevant given the widespread clinical use of gabapentinoids in neuropathic pain, underscoring that CIPNp represents a distinct clinical entity rather than a direct extension of other peripheral neuropathies.

Opioids are sometimes used in severe or refractory cases; however, their role in CIPNp is limited by modest efficacy, risk of adverse effects, and concerns regarding long-term use, particularly in cancer survivors [2]. Similarly, topical agents such as ketamine–amitriptyline creams have not demonstrated meaningful benefit, while high-concentration capsaicin patches have shown some efficacy in small studies, mainly in oxaliplatin-induced neuropathy, although their use remains limited by tolerability and accessibility issues [2].

Numerous nutraceuticals and supplements—including vitamin E, alpha-lipoic acid, glutathione, acetyl-L-carnitine, and omega-3 fatty acids—have been investigated for both prevention and treatment of CIPNp. To date, results have been inconsistent or negative, and some agents have even been associated with worsening neuropathic symptoms, highlighting the need for caution when considering empiric supplementation [2,6].

Taken together, the limited and inconsistent efficacy of pharmacological treatments reflects the multifactorial nature of CIPNp, in which ongoing peripheral nerve injury, central sensitization, neuroinflammation, and psychosocial factors interact to sustain pain. Pharmacological approaches primarily targeting isolated neurochemical pathways appear insufficient to address these complex and self-perpetuating mechanisms, reinforcing the need for alternative and integrative therapeutic strategies.

#### 3.3.2. Non-Pharmacological and Integrative Interventions

In light of the limited efficacy of pharmacological treatments, non-pharmacological and integrative interventions have gained increasing attention in the management of chemotherapy-induced peripheral neuropathic pain. These approaches aim to address functional impairment, pain persistence, and quality-of-life deterioration through mechanisms that extend beyond isolated neurochemical modulation, aligning more closely with the multifactorial pathophysiology of CIPNp [1,2].

Exercise-based rehabilitation is among the most consistently supported non-pharmacological strategies. Structured aerobic and resistance training programs have been shown to improve balance, gait stability, physical function, and, in some cases, neuropathic symptoms in patients with CIPNp. Proposed mechanisms include enhanced peripheral nerve perfusion, modulation of inflammatory pathways, improvement of mitochondrial function, and beneficial effects on central pain processing and psychological well-being [2,7]. Nevertheless, adherence may be limited in patients with severe pain, fatigue, or advanced functional impairment.

Cryotherapy and compression techniques, applied during chemotherapy infusion, have been investigated primarily as preventive strategies, particularly in taxane-based regimens. By reducing peripheral blood flow and limiting drug exposure to distal nerves, these interventions have demonstrated variable efficacy in reducing the incidence and severity of CIPN [2]. However, results remain inconsistent across studies, and their role in established CIPNp appears limited.

Neuromodulatory approaches, including transcutaneous electrical nerve stimulation (TENS), photobiomodulation, and other forms of peripheral or central stimulation, have also been explored. While preliminary findings suggest potential benefits in symptom reduction and nerve function, the current evidence base is heterogeneous and insufficient to support routine clinical implementation [2]. Methodological limitations, small sample sizes, and lack of standardized protocols remain significant barriers.

Psychological and behavioral interventions, such as cognitive–behavioral therapy and mindfulness-based approaches, may provide adjunctive benefit by targeting affective and cognitive components of chronic pain. Given the high prevalence of anxiety, depressive symptoms, sleep disturbance, and fear-avoidance behaviors in patients with chronic CIPNp, these interventions may contribute to improved coping and functional outcomes, although direct evidence specific to CIPNp is still limited [4].

Within this broad spectrum of non-pharmacological strategies, acupuncture has emerged as an integrative intervention with a growing body of evidence for CIPNp. Its generally favorable safety and tolerability profile, together with preliminary evidence suggesting possible benefits for neuropathic pain and other cancer-related symptoms, has contributed to growing interest in acupuncture within oncology supportive care settings [2,9,10]. Acupuncture has been proposed to influence several biological and psychosocial processes potentially relevant to pain, including peripheral inflammatory pathways, central pain modulation, autonomic regulation, and psychosocial dimensions of symptom perception. However, much of the mechanistic evidence is derived from animal studies or from neuropathic and chronic pain conditions other than CIPNp, and its direct relevance to CIPNp remains to be established. Current clinical evidence is also limited by small sample sizes and substantial heterogeneity in patient populations, acupuncture protocols, control conditions, outcome measures, and follow-up periods. Therefore, acupuncture cannot presently be regarded as a definitive or stand-alone treatment for CIPNp. Nevertheless, the available preliminary findings provide a rationale for further investigation of acupuncture as a potentially useful complementary component of individualized, integrative, and multimodal management strategies for CIPNp.

#### 3.3.3. Acupuncture for Chemotherapy-Induced Peripheral Neuropathic Pain: Clinical Evidence

Acupuncture is among the integrative interventions increasingly investigated for chemotherapy-induced peripheral neuropathy, including painful CIPN. Over the past decade, randomized controlled trials, pilot studies, and systematic reviews have examined its safety and potential clinical effects, contributing to growing interest in its possible role within oncology supportive care.

Early clinical studies reported possible reductions in neuropathic pain and sensory symptoms, as well as improvements in selected functional and quality-of-life outcomes. For example, the study by Lu et al. [15] provided preliminary evidence of the benefit of an acupuncture intervention compared with usual care, but it did not definitively establish the specific efficacy of acupuncture for painful chemotherapy-induced peripheral neuropathy or its superiority over sham acupuncture. Some subsequent systematic reviews and meta-analyses have also identified potentially favorable effects; however, their conclusions have not been consistent, and the overall certainty of the evidence remains limited [8,9,10]. In the review by Li et al. [16], including three randomized clinical trials, although two trials reported improvements in CIPN-related pain and quality of life, one found no significant benefit for neuropathic pain, other neuropathic symptoms, or quality of life. Because of the small evidence base, variable study quality, and moderate overall risk of bias, the authors concluded that the available evidence was insufficient to recommend acupuncture for the prevention or treatment of CIPN, although its use could be considered on an individual basis at the clinician’s discretion. Chien et al. [17] included six randomized controlled trials involving 386 patients with CIPN. Their meta-analysis identified statistically significant improvements in pain scores and patient-reported neurotoxicity symptoms, but no significant improvement in nerve conduction velocity. These findings suggest a possible symptomatic benefit; however, their interpretation is limited by the small number of trials, substantial heterogeneity in neuropathy-related outcomes, inconsistent outcome measures, and marked variation in control conditions and acupuncture protocols. Moreover, the included studies addressed CIPN broadly rather than exclusively pain-defined CIPNp. Accordingly, the results do not establish the efficacy of a specific acupuncture protocol or permit definitive conclusions regarding its effectiveness for CIPNp.

Recently, quantitative evidence syntheses have further examined the potential role of acupuncture in chemotherapy-induced peripheral neuropathy. A systematic review and meta-analysis by Zhao et al. [11] evaluated the efficacy and safety of acupuncture in cancer patients with chemotherapy-induced peripheral neuropathy. The pooled analysis reported statistically significant improvements in neuropathic pain severity, sensory symptoms, and overall neurological function in participants receiving acupuncture compared with the control interventions included in the analysis. Acupuncture was also generally well tolerated, and the reported adverse events were predominantly mild and transient. These findings suggest a possible therapeutic signal; however, they should not be interpreted as definitive evidence of efficacy, particularly for painful CIPN, because the included studies varied in their definitions, populations, outcome measures, and control conditions. Furthermore, pooled differences cannot, by themselves, establish that the observed effects are independent of placebo, expectancy, or other nonspecific treatment effects. In this context, the study by Bao et al. [18] is particularly relevant: the authors observed greater reductions in pain, tingling, and numbness with real acupuncture, particularly when compared with usual care. At eight weeks, the mean reduction in pain severity was 1.75 points in the real acupuncture group, 0.91 points in the sham acupuncture group, and 0.19 points in the usual-care group. Adverse events were infrequent and mild. However, the authors noted that this was a small, single-center pilot study with a short follow-up period. It did not definitively establish the superiority of real acupuncture over sham acupuncture. Moreover, it included patients with symptomatic CIPN, assessed based on pain, numbness, or tingling, rather than exclusively patients with painful CIPN, defined by the presence of pain.

More recently, Xu et al. [19] provided additional sham-controlled evidence in a single-center, randomized, single-blind trial. Seventy patients with CIPN were randomized to verum or sham acupuncture, with both groups also receiving oral mecobalamin; 68 patients were included in the final analysis. After 2 weeks, verum acupuncture was associated with greater improvements in NCI-CTCAE grade, EORTC QLQ-CIPN20 and NRS pain scores, as well as in selected nerve conduction parameters. No serious adverse events were reported. Although the sham-controlled design strengthens the assessment of a treatment-specific effect, interpretation remains limited by the small single-center sample and the very short intervention and follow-up periods. These findings therefore provide supportive but not definitive evidence for acupuncture in CIPN, and their applicability specifically to pain-defined CIPNp requires further confirmation.

At the evidence-synthesis level, Yeh et al. [20] conducted an umbrella review of 14 systematic reviews and reported generally favorable findings for acupuncture-related interventions, including manual acupuncture, electroacupuncture, and transcutaneous electrical acupoint stimulation, across CIPN symptoms, pain, nerve conduction measures, and quality of life. The included reviews were reported to have moderate-to-high methodological and reporting quality. Nevertheless, because an umbrella review synthesizes previously published systematic reviews that may include overlapping primary trials, these findings are best interpreted as supporting the consistency of the overall therapeutic signal rather than as independent confirmation of acupuncture efficacy, particularly for pain-defined CIPNp.

Notably, Zhao et al. [11] also highlighted substantial heterogeneity across the included trials, particularly with respect to acupuncture modality (manual acupuncture, electroacupuncture, auricular acupuncture), point selection, treatment frequency and duration, outcome measures, and control conditions. Similar variability has been reported in earlier reviews and limits direct comparison between studies, reduces confidence in the pooled estimates, and prevents the definition of standardized treatment protocols. Although this heterogeneity may partly reflect differences in clinical practice and treatment individualization, it remains an important methodological limitation. Accordingly, the findings support further the need for further well-designed, adequately powered trials using standardized reporting, clearly defined pain-specific outcomes, appropriate control groups, and longer follow-up periods.

The potential relevance of treatment dose was specifically examined by Tian et al. [21] in a dose–response meta-analysis of 11 randomized controlled trials involving 740 participants. Pooled analyses suggested reductions in pain and improvements in quality-of-life outcomes, but heterogeneity for the pain outcome was substantial (I^2^ = 95%). Nonlinear modelling suggested that clinically meaningful pain improvement may occur at approximately 16 treatment sessions delivered about twice weekly over 8 weeks, at a frequency of about twice weekly. Importantly, subgroup analysis did not show a statistically significant difference in pain when acupuncture was compared specifically with sham acupuncture. Accordingly, the proposed temporal parameters should be regarded as exploratory and hypothesis-generating rather than as an established optimal treatment regimen.

Complementing these dose–response observations, Shen et al. [22] directly compared three electroacupuncture frequencies (2 Hz, 100 Hz, and 2/100 Hz) with mecobalamin in a single-center, participant-blinded, four-arm randomized pilot trial in breast cancer patients with CIPN. The 2/100 Hz group showed the highest overall response rate, whereas 2 Hz electroacupuncture showed advantages for sensory symptoms; different frequency groups also showed distinct profiles across selected quality-of-life domains. Nevertheless, the study did not include a sham electroacupuncture group, relied predominantly on patient-reported outcomes, and included relatively small groups. In addition, pain-specific efficacy could not be accurately evaluated because some participants used analgesic medication. The findings therefore do not establish frequency-specific efficacy or define an optimal stimulation frequency, but they reinforce the hypothesis that treatment parameters may contribute to heterogeneity in clinical response and warrant prospective evaluation.

From a clinical perspective, favorable findings reported across different acupuncture-based interventions suggest a potential therapeutic signal in CIPN and, more specifically, in painful CIPN. In this context, differences in treatment response by neuropathic phenotype, chemotherapeutic agent, or underlying pathophysiological mechanisms remain plausible hypotheses, but they have not been consistently demonstrated in clinical trials and require further investigation.

Overall, the current clinical evidence suggests that acupuncture is generally well tolerated and may provide symptomatic benefit for some patients with CIPN or CIPNp. Nevertheless, the heterogeneity of the available studies, the small sample sizes, the variability in control conditions, the limited duration of follow-up, and the inconsistent use of pain-specific outcomes preclude definitive conclusions regarding its efficacy. Acupuncture may therefore be considered a complementary option within individualized and multidisciplinary supportive care, particularly when conventional symptom-management strategies provide insufficient relief, provided that patient preferences, clinical circumstances, and potential limitations of the evidence are discussed. Further adequately powered trials using standardized reporting, appropriate control groups, clearly defined CIPNp outcomes, and longer follow-up are needed before acupuncture can be recommended as a routine or core component of CIPNp management. Table 5 summarizes the findings for the different therapeutic approaches to CIPNp reviewed in this article.

#### 3.3.4. Mechanisms of Acupuncture in Chemotherapy-Induced Peripheral Neuropathic Pain

A growing body of evidence suggests that acupuncture may act through multiple complementary mechanisms operating at the peripheral, spinal, supraspinal, autonomic, and neuroimmune levels. This multimodal action could be particularly relevant in CIPNp, a condition characterized by the interaction of ongoing peripheral nerve injury, central sensitization, neuroinflammation, and psychosocial contributors, as outlined in the 3P etiological model. The 3P model (predisposing, precipitating, and perpetuating factors) is widely recognized as a robust conceptual framework for understanding complex clinical conditions. Wright et al. [23] describe the application of the 3P model across various health contexts, including chronic pain, gastrointestinal disorders, oral diseases, and cardiovascular diseases. A more recent study by Azizoddin et al. [24] proposes the 3P-CP model specifically for cancer pain, conceptualizing cancer-related pain through interconnected phases that enable a structured assessment across the entire cancer trajectory. The mechanisms discussed in this section should be interpreted primarily as evidence of biological plausibility rather than as confirmed mediators of clinical benefit in patients with CIPNp. Direct human mechanistic evidence in CIPNp remains limited. A substantial proportion of the available evidence derives from experimental animal models of chemotherapy-induced neuropathy or from human studies involving other neuropathic and chronic pain conditions.

Peripheral and spinal mechanisms

At the peripheral level, acupuncture has been shown to modulate nociceptive signaling through activation of large-diameter afferent fibers, consistent with gate control theory, thereby inhibiting transmission of nociceptive input from C and Aδ fibers at the spinal dorsal horn [25,26]. This mechanism could be particularly relevant in CIPNp, where peripheral nerve hyperexcitability and spontaneous ectopic discharges contribute to ongoing pain. In addition, some studies suggest that acupuncture may influence the local neurochemical milieu by reducing the release of pro-inflammatory cytokines and chemokines and by modulating the expression and function of ion channels implicated in neuropathic pain, including sodium and calcium channels [26,27]. Although these findings require confirmation in clinical mediation studies in CIPNp, these effects may attenuate peripheral sensitization and reduce ongoing nociceptive input arising from chemotherapy-damaged nerves.

Endogenous analgesic systems and descending modulation

Acupuncture has been proposed to modulate endogenous analgesic systems through changes in neurotransmitters and neuromodulators, including endogenous opioids (endorphins, enkephalins), serotonin, and noradrenaline. These effects may influence descending inhibitory pathways from supraspinal centers to the spinal cord, contributing to pain modulation [26]. Although some clinical studies have reported reductions in pain intensity and improvements in pain-related outcomes following acupuncture, these observations do not by themselves confirm that the proposed neurochemical mechanisms are responsible for the clinical effects or that they operate similarly in patients with CIPNp.

Neuroimmune and anti-inflammatory effects

Neuroinflammation is thought to contribute to the development and persistence of CIPNp, particularly in taxane- and proteasome inhibitor-induced neuropathies [2,27]. Experimental studies suggest that acupuncture may modulate inflammatory signaling, including cytokine profiles and regulation of microglial and astrocytic responses. These effects could potentially influence neuroimmune mechanisms involved in peripheral and central sensitization. However, much of the supporting evidence derives from animal models or from neuropathic pain conditions other than CIPNp.

Autonomic and vagal modulation

Autonomic dysregulation has been proposed as a potential contributor to some chronic pain conditions, although its specific role in CIPNp remains insufficiently characterized. Experimental and clinical studies conducted in other populations suggest that acupuncture may influence autonomic activity, including parasympathetic and sympathetic regulation. Modulation of vagal activity has also been hypothesized to affect inflammatory signaling through the cholinergic anti-inflammatory pathway [26]. These mechanisms could potentially contribute to pain modulation; however, direct evidence demonstrating their relevance to acupuncture-related clinical outcomes in patients with CIPNp is currently limited.

Central neuroplasticity and network modulation

Beyond these peripheral and spinal mechanisms, emerging evidence suggests that acupuncture may also influence functional and, potentially, structural features of central pain-processing networks. Functional neuroimaging studies have reported changes in activity and connectivity within large-scale brain networks involved in sensory processing, affective regulation, and cognitive control, including the sensorimotor network, frontoparietal executive network, limbic structures, and the default mode network [27,28]. These findings may be relevant to CIPNp, in which persistent pain has been associated not only with ongoing peripheral nerve damage but also with central sensitization and maladaptive neuroplastic changes [2,3]. It is therefore plausible that modulation of altered network connectivity and neural processing may contribute to some of the clinical effects associated with acupuncture. However, the extent to which these central changes mediate sustained symptom improvement remains uncertain and requires further investigation. Table 6 summarizes the main mechanisms proposed to underline the potential effects of acupuncture in CIPNp.

The potential multimodal mechanisms of acupuncture may help explain some of the variability in therapeutic response observed across CIPNp subtypes. Neuropathies predominantly driven by neuroinflammation and peripheral sensitization, such as taxane-induced CIPNp, might theoretically respond differently from platinum-induced neuropathies characterized by dorsal root ganglion toxicity and more persistent structural neuronal damage [2,29,30]. However, current evidence is insufficient to establish whether the underlying chemotherapeutic agent or neuropathic phenotype reliably predicts the response to acupuncture. This mechanistic framework may nevertheless provide a basis for future phenotype-informed studies and is broadly consistent with the clinical heterogeneity summarized in Table 4.

Taken together, the available evidence suggests that acupuncture may influence several mechanisms potentially involved in the persistence of CIPNp rather than acting through a single neurochemical pathway. These may include peripheral nerve function, central sensitization, neuroimmune activity, autonomic balance, and brain network connectivity. Such potentially broad effects provide a biologically plausible rationale for investigating acupuncture as a complementary treatment for CIPNp. Although recent meta-analyses, including that by Zhao et al. [11], have reported beneficial clinical effects, the certainty and consistency of the evidence remain limited, and further well-designed studies are needed to clarify the mechanisms involved, identify potential predictors of response, and determine the magnitude and durability of any clinical benefit.

#### 3.3.5. Chemotherapy Class and Clinical Phenotype: Hypothesis-Generating Considerations

CIPNp is increasingly recognized as a heterogeneous clinical condition rather than a uniform neuropathic entity. Differences in underlying neurotoxic mechanisms, fiber involvement, inflammatory burden, and central sensitization contribute to distinct clinical phenotypes and may influence therapeutic response. Recognizing this heterogeneity is essential for optimizing treatment selection and for interpreting the variability observed across clinical trials.

As summarized in Table 2, platinum-based compounds, particularly oxaliplatin, are associated with a predominantly sensory neuropathy characterized by dorsal root ganglion toxicity, cold-induced allodynia, and marked peripheral nerve hyperexcitability. In these patients, neuropathic pain may coexist with persistent sensory loss and altered thermal perception. In contrast, taxane-induced CIPNp is strongly associated with neuroinflammatory processes, distal axonal degeneration, and peripheral sensitization. Clinically, this phenotype often presents with burning pain, dysesthesia, and functional impairment, with a comparatively lower contribution of dorsal root ganglion toxicity.

Neuropathies induced by proteasome inhibitors, such as bortezomib, represent a distinct and less well-studied phenotype. These neuropathies often involve small-fiber dysfunction and prominent pain, but data regarding acupuncture efficacy in this subgroup remain limited. The scarcity of targeted studies highlights an important gap in the literature and underscores the need for phenotype-specific investigations [2].

Beyond drug class, the relative contribution of perpetuating factors—such as central sensitization, affective dysregulation, sleep disturbance, and fear-avoidance behaviors—may further influence therapeutic response. Patients with prominent central features, including disproportionate pain severity relative to peripheral nerve damage, may derive particular benefit from acupuncture due to its capacity to modulate central pain-processing networks, as suggested by functional neuroimaging studies [27,28].

Acupuncture-associated symptom improvements have been reported across heterogeneous CIPN populations. However, current data do not establish whether treatment response differs according to neuropathic phenotype or the relative contribution of inflammatory or central sensitization mechanisms.

Available studies have included patients exposed to taxanes, platinum compounds, and other neurotoxic agents. However, current evidence does not establish whether acupuncture efficacy differs by chemotherapy class or neuropathic phenotype, because comparisons are based mainly on separate studies using different eligibility criteria, acupuncture protocols, control interventions, outcome measures, and follow-up periods.

The distinct pathophysiological characteristics of taxane- and platinum-induced neuropathies provide a rationale for future stratified analyses. Consequently, chemotherapy class and clinical phenotype should currently be considered variables for stratification in future trials. Future studies should report outcomes separately by neurotoxic agent, predominant sensory phenotype, pain characteristics, baseline neurological impairment, and duration of symptoms.

### 3.4. Neuroimaging and Biomarker Perspectives in Chemotherapy-Induced Peripheral Neuropathic Pain

The absence of validated objective biomarkers remains a major limitation for the diagnosis, phenotypic stratification, and treatment monitoring of chemotherapy-induced peripheral neuropathic pain. Current assessment relies predominantly on patient-reported outcomes and clinical examination, which, although essential, may inadequately capture central nervous system contributions to persistent pain and treatment response. This gap has stimulated growing interest in neuroimaging approaches, particularly functional magnetic resonance imaging (fMRI), as potential tools for mechanistic investigation and biomarker development in CIPNp.

Functional neuroimaging studies in chronic neuropathic pain conditions have consistently demonstrated alterations in large-scale brain networks involved in sensory processing, affective regulation, and cognitive control, including the sensorimotor network, limbic circuitry, frontoparietal executive network, and the Default Mode Network. Although data specific to CIPNp remain limited, emerging evidence suggests that similar patterns of maladaptive connectivity and central sensitization may contribute to symptom persistence in this population.

In the context of acupuncture, fMRI offers a unique opportunity to investigate treatment-related neuroplasticity. Studies across various chronic pain conditions have shown that acupuncture modulates functional connectivity within pain-related brain networks, normalizes aberrant network interactions, and engages descending pain modulatory systems. These neurofunctional changes often parallel clinical improvements in pain intensity, sensory symptoms, and affective distress, supporting a plausible link between central network modulation and therapeutic response. Applied to CIPNp, fMRI-based assessments may serve as exploratory biomarkers to characterize baseline neurofunctional phenotypes, identify patients with prominent central sensitization, and monitor neural correlates of response to acupuncture. Such an approach aligns with the observed heterogeneity of CIPNp and may help explain differential treatment outcomes across chemotherapeutic agents and clinical phenotypes, as discussed in the previous section.

Importantly, fMRI should currently be regarded as a research tool rather than a diagnostic or predictive biomarker for routine clinical use. Limitations related to cost, accessibility, inter-individual variability, and lack of standardized protocols preclude immediate clinical translation. Nevertheless, integration of fMRI into prospective clinical trials may provide critical mechanistic insights, facilitate biomarker discovery, and support the development of mechanism-informed, personalized treatment strategies for CIPNp.

By bridging subjective symptom reports with objective measures of central nervous system function, neuroimaging approaches such as fMRI represent a promising avenue for advancing the understanding and management of chemotherapy-induced peripheral neuropathic pain, particularly in the evaluation of integrative interventions such as acupuncture.

## 4. Discussion

By integrating epidemiological data, etiological mechanisms, diagnostic challenges, and therapeutic evidence, the present work underscores the multifactorial nature of CIPNp and the limitations of current conventional treatment paradigms. The available evidence suggests a possible clinical signal for acupuncture in the symptomatic management of CIPN and CIPNp; however, the evidence remains insufficient to establish definitive clinical efficacy. Positive findings reported in individual trials and pooled analyses should therefore be interpreted in the context of small sample sizes, clinical and methodological heterogeneity, variable control interventions, and limited follow-up.

Because this was a non-systematic narrative review, the findings should be understood as an integrative and conceptually oriented synthesis of selected literature relevant to the topic rather than as an exhaustive or quantitatively pooled evaluation of the evidence. No predefined review protocol, formal risk-of-bias assessment, or evaluation of the certainty of evidence was undertaken. Consequently, the possibility of selective literature inclusion cannot be excluded. Moreover, although the present review focuses on CIPNp, several studies included patients with CIPN more broadly and assessed composite neurological or sensory outcomes rather than pain-specific endpoints. Evidence concerning CIPN should therefore not automatically be interpreted as evidence specifically supporting an effect on painful CIPN.

The clinical literature provides mixed and preliminary findings. Randomized studies, including those by Bao et al. [18] and Lu et al. [15] as well as the more recent studies by Xu et al. [19] and Shen et al. [22], provide additional support for a possible clinical signal that acupuncture-based interventions may influence CIPN symptoms, but their small sample sizes, predominantly single-center design, limited follow-up and, in the case of Shen et al., absence of a sham control, do not resolve uncertainty regarding specific efficacy, durability, optimal treatment parameters, or applicability to pain-defined CIPNp. Evidence syntheses such as those by Li et al. [16], Chien et al. [17], and Zhao et al. [11] should be considered together because they illustrate differences in study design, outcome selection, methodological quality, and interpretation of the evidence. Recent higher-level evidence syntheses provide additional support for a possible therapeutic signal. The umbrella review by Yeh et al. [20] found broadly favorable findings across several CIPN-related outcomes, whereas the dose–response meta-analysis by Tian et al. [21] suggested that treatment frequency and duration may influence clinical response. However, these findings do not resolve the major limitations of the evidence base, including potential overlap among primary studies included in successive reviews, substantial between-study heterogeneity, and uncertainty regarding the specific effect of acupuncture when compared with credible sham interventions. Although some trials and pooled analyses have reported improvements in pain, sensory symptoms, or quality of life, other reviews have concluded that the available evidence is insufficient to support firm recommendations for either the prevention or treatment of CIPN. Accordingly, statistically significant pooled findings should not be interpreted in isolation from the quality and comparability of the underlying studies.

Important methodological limitations include small sample sizes, variability in acupuncture modality and treatment dose, differences in chemotherapy exposure and patient characteristics, inconsistent definitions of CIPN and CIPNp, heterogeneous outcome measures, and short or absent post-treatment follow-up. The design of appropriate control conditions also remains challenging. Sham acupuncture may produce physiological or expectancy-related effects and is not necessarily an inert comparator, whereas comparisons with usual care do not adequately control for attention, patient expectations, or other contextual effects. Difficulties in blinding participants and practitioners further complicate the attribution of observed improvements to acupuncture-specific mechanisms. Together, these limitations reduce confidence in effect estimates and prevent reliable identification of patients who may be more likely to benefit.

The 3P framework may nevertheless offer conceptual value by distinguishing predisposing, precipitating, and perpetuating components of CIPNp. Patient- and treatment-related vulnerabilities may predispose individuals to neuropathy, while chemotherapy-induced neural injury represents the principal precipitating event. Persistent symptoms may subsequently be maintained by interacting with peripheral, spinal, central, neuroimmune, autonomic, and psychosocial processes. Within this framework, acupuncture may be more appropriately considered a potential modifier of some perpetuating mechanisms and associated symptoms rather than an intervention that reverses the initiating neurotoxic injury. This distinction helps avoid attributing disease-modifying effects to acupuncture in the absence of supporting evidence.

Several biological mechanisms could plausibly contribute to acupuncture-associated symptom changes, including modulation of peripheral nerve function, spinal nociceptive processing, neuroimmune activity, endogenous pain-regulatory systems, autonomic function, and central pain-processing networks. However, the evidence base for these mechanisms is uneven. Some observations derive from clinical studies of CIPN, whereas much of the mechanistic evidence has been extrapolated from animal experiments or from other neuropathic and chronic pain conditions. These sources may support biological plausibility but do not demonstrate that the same mechanisms operate in patients with CIPNp or mediate clinical improvement. Accordingly, a consistent distinction should be maintained between mechanisms directly investigated in CIPN or CIPNp and those inferred from related experimental or clinical pain models. Similarly, differences between taxane- and platinum-associated neuropathies may provide a rationale for future mechanistic and stratified research, but current evidence does not establish that the causative chemotherapeutic agent reliably predicts response to acupuncture. Taxane-related neuroinflammation, platinum-associated dorsal root ganglion toxicity, and other agent-specific mechanisms may theoretically influence treatment responsiveness. It may be hypothesized that taxane- and platinum-associated neuropathies differ in their responsiveness to acupuncture; however, this possibility has not been established clinically and should currently be examined only through prospective, appropriately stratified studies. At present, these distinctions should inform study design rather than clinical treatment selection.

Functional neuroimaging may also contribute to future mechanistic studies by identifying neural correlates of persistent pain and treatment-related changes. Nevertheless, fMRI is not currently validated as a diagnostic, prognostic, or predictive biomarker in CIPNp. Observed changes in brain activity or connectivity should be regarded as an exploratory translational tool that may complement rigorous clinical outcomes and appropriately controlled trial designs.

Current evidence does not support positioning acupuncture as a core component of standard CIPNp management. Where available, it may be discussed as a complementary option for selected patients within a multidisciplinary and shared decision-making framework, particularly when conventional symptom-management approaches provide insufficient relief. Such discussions should include the preliminary nature of the evidence, uncertainty regarding the magnitude and durability of benefit, and the lack of validated predictors of response.

Future trials should be adequately powered and prospectively registered, clearly distinguish CIPN from pain-specific CIPNp, and define pain as a primary outcome when the therapeutic objective is pain reduction. Greater consistency is also needed in eligibility criteria, acupuncture procedures, treatment dose, comparator selection, outcome instruments, adverse-event reporting, and duration of follow-up. Wider use of the STRICTA recommendations [31] would improve the transparency and reproducibility of acupuncture reporting, although STRICTA compliance alone would not resolve problems related to bias, heterogeneity, or inadequate statistical power. Future studies could also prespecify stratification by chemotherapeutic agent, neuropathic phenotype, and features of central sensitization, while incorporating mechanistic assessments and exploratory neuroimaging where justified.

In summary, the available literature suggests that acupuncture may provide symptomatic benefit for some patients with CIPN or CIPNp, but the current evidence remains heterogeneous and insufficient to establish definitive efficacy or support routine clinical use. The main contribution of the present review lies in integrating clinical observations with the 3P framework and potential peripheral and central mechanisms, while explicitly recognizing the largely hypothesis-generating nature of several mechanistic and phenotype-specific interpretations. More rigorous and transparently reported clinical research is required to determine whether acupuncture provides clinically meaningful and durable benefits, to identify potential responders, and to clarify its appropriate place within multimodal CIPNp management.

## 5. Conclusions

CIPNp represents a prevalent and challenging complication of contemporary cancer treatment, with substantial impact on long-term functioning and quality of life. Its multifactorial etiology, heterogeneous clinical presentation, and frequent persistence beyond chemotherapy completion underscore the limitations of conventional pharmacological approaches and highlight the need for integrative, mechanism-informed management strategies.

Current evidence suggests that acupuncture may be associated with improvements in pain and sensory symptoms in some patients with CIPN or CIPNp and appears to be generally well tolerated in the studies conducted to date. However, small sample sizes, heterogeneous patient populations and acupuncture protocols, variable control conditions and outcome measures, difficulties in blinding, and limited follow-up preclude definitive conclusions regarding efficacy, durability of benefit, or routine clinical use.

The main contribution of this narrative review lies in integrating the available clinical evidence with the 3P framework and potential peripheral and central mechanisms of symptom persistence. Within this framework, acupuncture may be more appropriately regarded as a possible modifier of selected perpetuating mechanisms and associated symptoms rather than as an intervention capable of reversing the initial chemotherapy-induced neurotoxic injury. Although the proposed neuroimmune, neurochemical, autonomic, and central mechanisms provide biological plausibility, much of the supporting evidence derives from animal models or non-CIPN pain conditions and should therefore be considered hypothesis-generating.

At present, acupuncture should not be positioned as a core component of standard CIPNp management. It may be discussed as a monitored complementary option for selected patients within individualized, multidisciplinary, and shared decision-making care, particularly when conventional symptom-management strategies provide insufficient relief. Future trials should be adequately powered and prospectively registered, distinguish CIPN from pain-defined CIPNp, use pain-specific and clinically meaningful outcomes, employ appropriate control conditions and standardized, STRICTA-compliant reporting, and include longer follow-up. Prospective stratification by chemotherapeutic agent and neuropathic phenotype, together with mechanistic assessments and exploratory neuroimaging, may help clarify potential treatment-response modifiers and the appropriate role of acupuncture in multimodal CIPNp management.

## Figures and Tables

**Table 1:** Prevalence and clinical frequency of chemotherapy-induced peripheral neuropathy (CIPN) across major antineoplastic drug classes and targeted cancer types, adapted from Banach et al. [5].

Antineoplastic Agent Class/Drug	Cancer Types Indicated	Estimated CIPN Prevalence Rate	Clinical CIPN Frequency
Platinum Derivatives	Ovarian, lung, colorectal, bladder, testicular germ cell	70–100%	Very High
Taxanes	Breast, lung, ovarian, pancreatic, prostate	11–87%	High
Ixabepilone	Breast	60–65%	High
Thalidomide & Analogues	Multiple myeloma	20–60%	High
Bortezomib	Multiple myeloma	20–30%	Moderate
Vinca Alkaloids	Lung, bladder, brain, testicular	Up to 20%	Moderate

**Table 2:** Mechanisms of action and clinical presentations of neurotoxicity associated with major classes of antineoplastic agents, adapted from Bonomo & Cavaletti [3].

Antineoplastic Drug Class	Key Mechanisms of Neurotoxicity	Clinical Features & Presentation
Platinum Derivatives	Irreversible nuclear and mitochondrial DNA (mDNA) adducts impairing replication/transcription; extracellular Ca^2+^ chelation and membrane potential disruption (oxaliplatin); altered expression of Ca_v_2.2 and TRP ion channels; glial cell activation driving neuroinflammation	Sensory neuropathies (numbness, paresthesias, dysesthesias, impaired proprioception); acute cold-induced paresthesias (oxaliplatin)
Vinca Alkaloids	Binding to β-tubulin subunits leading to mitotic inhibition; disruption of intracellular Ca^2+^ dynamics and neuronal hyperexcitability triggering apoptotic signaling	Distal motor weakness and autonomic nervous system dysfunction
Taxanes (e.g., *Paclitaxel*, *Docetaxel*)	Dysregulation of tubulin polymerization; mitochondrial Ca^2+^ efflux causing membrane depolarization; down-regulation of K^+^ channels; elevation of pro-inflammatory cytokines TNF-α, IL-1β) promoting neuroinflammation	Predominantly sensory deficits (neuropathic pain, hypoesthesia); motor and autonomic involvement (less frequent)
Proteasome Inhibitors	Interaction with α-tubulin disrupting cytoskeletal organization; upregulation of sphingolipid pathways generating elevated S1P and DH-S1P; spinal cord dorsal horn glutamate release via glial S1PR1 signaling	Sensory neuropathies, predominantly presenting as neuropathic pain
Immune Checkpoint Inhibitors	Skewed T-cell lineage differentiation toward Th1/Th17 pro-inflammatory profiles; suppression of Th2 cytokines (IL-5, IL-13); antibody-mediated toxicity and complement cascade activation	Mixed sensory and motor neuropathic manifestations

**Table 3:** Time-course prevalence and drug-specific characteristics of chemotherapy-induced peripheral neuropathy (CIPN) following completion of treatment, adapted from Seretny [1] and Bonomo & Cavaletti [3].

Post-Chemotherapy Follow-Up Period	CIPN/NPIQ Prevalence Rate
Short-term (≤1 month post-treatment)	68.1%
Mid-term (3 months post-treatment)	60.0%
Long-term (≥6 months post-treatment)	30.0%

**Table 4:** Diagnostic tools, patient-reported outcome measures, and objective clinical examination methods for chemotherapy-induced peripheral neuropathy (NPIQ/CIPN), adapted from Burgess et al. [2] and Bonomo & Cavaletti [3].

Assessment Modality	Domain/Subcategory	Specific Tools and Diagnostic Clinical Tests
Patient-Reported Outcomes & Questionnaires (incorporating CTC scales)	Neurotoxicity Assessment	FACT-GOG-Ntx (Functional Assessment of Cancer Therapy/Gynecologic Oncology Group—Neurotoxicity)
		CIPNAT (Chemotherapy-Induced Peripheral Neuropathy Assessment Tool)
	Quality of Life (QoL)	EORTC QLQ-C30 (European Organisation for Research and Treatment of Cancer Core Quality of Life Questionnaire)
		SF-36 (36-Item Short Form Health Survey)
Comprehensive Clinical Examination	Objective Neurological Testing	Evaluation of deep tendon reflexes
		Quantitative sensory testing for vibration perception threshold
		Light touch and pinprick tactile sensitivity
		Motor strength grading via Medical Research Council (MRC) criteria
		Electrodiagnostic nerve conduction studies (NCS)

**Table 5:** Results reported from different therapeutic approaches to chemotherapy-induced peripheral neuropathic pain.

Study	Intervention/Focus	Type of Evidence	Main Findings
Seretny et al. (2014) [1]	Epidemiology, clinical burden, and therapeutic limitations of chemotherapy-induced peripheral neuropathy and neuropathic pain	Review/evidence synthesis	Highlights the high prevalence and clinical impact of CIPN, as well as the lack of consistently effective preventive pharmacological strategies.
Hou et al. (2018) [13]	Pharmacological treatments commonly extrapolated from other neuropathic pain conditions	Review/clinical evidence	Reports inconsistent or disappointing efficacy for several pharmacological classes, including antidepressants and anticonvulsants, when applied to CIPNp.
Loprinzi et al. (2020) [6]	Pharmacological management of CIPNp	Guideline/clinical review	Identifies duloxetine as the pharmacological agent with the strongest evidence, although its benefit is modest. Other agents, including gabapentinoids and tricyclic antidepressants, show limited or inconsistent efficacy.
Burgess et al. (2021) [2]	Pharmacological and non-pharmacological interventions for CIPN/CIPNp	Review	Summarizes the limited efficacy of pharmacological agents and discusses non-pharmacological strategies, including exercise, cryotherapy, compression, neuromodulation, topical agents, supplements, and acupuncture.
Chow et al. (2022) [14]	Duloxetine for chemotherapy-induced peripheral neuropathic pain	Clinical evidence/randomized trials as referenced in the text	Supports a modest reduction in pain severity with duloxetine, particularly in oxaliplatin-induced neuropathy, although treatment response appears heterogeneous and tolerability may be limited by adverse effects.
Oh and Kim (2018) [7]	Exercise-based rehabilitation	Review/clinical evidence	Structured aerobic and resistance exercise may improve balance, gait stability, physical function, and, in some cases, neuropathic symptoms. Proposed mechanisms include improved nerve perfusion, inflammatory modulation, mitochondrial effects, and central pain modulation.
Capela et al. (2023) [4]	Psychological and behavioral interventions	Evidence specific to chronic pain-related psychosocial factors; limited direct evidence in CIPNp	Cognitive–behavioral and mindfulness-based approaches may improve coping, emotional distress, sleep disturbance, and functional outcomes, although direct evidence in CIPNp remains limited.
Franconi et al. (2013) [9]	Acupuncture for neuropathic pain and cancer-related symptoms	Early clinical evidence/review	Suggests that acupuncture may alleviate neuropathic pain, reduce sensory symptoms, and improve functional outcomes, with a favourable safety profile.
Dimitrova et al. (2017) [10]	Acupuncture for chemotherapy-induced peripheral neuropathy	Systematic review/clinical evidence	Reported potential benefits of acupuncture in reducing neuropathic symptoms and improving function, while acknowledging methodological variability across studies.
Hwang et al. (2020) [8]	Acupuncture for CIPN/CIPNp	Systematic review and/or meta-analysis	Provides additional evidence that acupuncture may be associated with clinically meaningful reductions in pain intensity and sensory dysfunction, alongside improvements in quality of life.
Zhao et al. (2025) [11]	Acupuncture in cancer patients with chemotherapy-induced peripheral neuropathy	Systematic review and meta-analysis	Pooled analyses suggested statistically significant improvements in neuropathic pain severity, sensory symptoms, and neurological function compared with control interventions. Adverse events were generally mild and transient. The review also identifies heterogeneity in acupuncture modality, point selection, treatment frequency, duration, outcome measures, and controls.
Lu et al. (2020) [15]	Acupuncture for persistent taxane-induced CIPN in breast cancer survivors	Randomized controlled pilot trial	Reported improvements in sensory symptoms, neurotoxicity-related quality of life, and pain severity after 8 weeks of acupuncture compared with usual care. The small sample size, wait-list control design, and restriction to breast cancer survivors treated with taxanes limit generalizability and do not establish efficacy specifically for CIPNp.
Li et al. (2019) [16]	Acupuncture for prevention and treatment of CIPN	Systematic review	Included three randomized trials involving 203 participants. Two studies reported improvements in CIPN-related pain and quality of life, whereas one found no benefit. Because of the small evidence base, variable methodological quality, and moderate overall risk of bias, the authors considered the evidence insufficient to recommend acupuncture for CIPN.
Chien et al. (2019) [17]	Acupuncture for chemotherapy-induced peripheral neuropathy	Systematic review and meta-analysis	Included six randomized trials involving 386 patients. Pooled analyses suggested improvements in pain and patient-reported neurotoxicity symptoms, but not in nerve conduction velocity. The limited number of studies, heterogeneity in interventions and controls, and variability in outcome measures restrict the certainty and generalizability of the findings.
Bao et al. (2020) [18]	Acupuncture for chemotherapy-induced peripheral neuropathy symptoms	Randomized controlled trial	Compared real acupuncture with sham acupuncture and usual care. Real acupuncture was associated with greater reductions in pain, tingling, and numbness, particularly compared with usual care. However, the small sample size and limited differences between real and sham acupuncture preclude definitive conclusions regarding specific efficacy for CIPNp.
Xu et al. (2025) [19]	Verum acupuncture + mecobalamin vs. sham acupuncture + mecobalamin in patients with CIPN	Randomized, single-center, sham-controlled trial	Seventy patients were randomized and 68 analyzed. After 2 weeks, verum acupuncture was associated with greater improvement in NCI-CTCAE neuropathy grade, EORTC QLQ-CIPN20, NRS pain, and selected nerve-conduction parameters; no serious adverse events were reported. The short treatment/follow-up, small single-center sample, concomitant mecobalamin, and TCM syndrome-based eligibility limit generalizability and durability of the findings.
Yeh et al. (2025) [20]	Acupuncture-related interventions for CIPN symptoms, pain, and quality of life	Umbrella review of systematic reviews	Synthesized 14 systematic reviews and reported generally favorable findings for acupuncture-related interventions across CIPN symptoms, pain, nerve conduction measures, and quality of life. Because an umbrella review synthesizes previously published reviews that may contain overlapping primary trials, these findings strengthen the overall therapeutic signal but should not be regarded as independent confirmation of efficacy, particularly for pain-defined CIPNp.
Tian et al. (2025) [21]	Acupuncture treatment dose and temporal parameters for CIPN	Systematic review and dose–response meta-analysis	Included 11 randomized controlled trials involving 740 participants. Pooled analyses suggested reductions in pain and improvements in quality-of-life outcomes, but heterogeneity for pain was substantial (I^2^ = 95%). Dose–response modelling suggested that clinically meaningful pain improvement may occur at approximately 16 sessions over 8 weeks, at a frequency of twice weekly. In the subgroup using sham acupuncture as the comparator, the difference in pain was not statistically significant; therefore, the proposed temporal parameters should be considered exploratory rather than an established optimal regimen.
Shen et al. (2025) [22]	Electroacupuncture frequency comparison (2 Hz, 100 Hz, 2/100 Hz) vs. mecobalamin in breast-cancer patients with CIPN	Four-arm randomized, participant-blinded pilot trial	One hundred sixty patients were randomized and 152 included in modified intention-to-treat analysis. The 2/100 Hz group showed the highest overall response rate; 2 Hz and 2/100 Hz showed improvements in sensory symptoms and different quality-of-life domains, whereas 100 Hz showed no comparable benefit. Single-center design, absence of sham EA, predominantly patient-reported outcomes, and analgesic use make frequency-specific conclusions exploratory.

**Table 6:** Mechanistic pathways, biological effects, and clinical relevance in CIPNp.

Underlying Mechanism	Key Biological Effects	Clinical Relevance in CIPNp
Peripheral & Spinal Modulation	Activation of large-diameter myelinated afferent nerve fibers, inhibiting nociceptive transmission at the spinal dorsal horn (Gate Control Theory).	Dampens primary nociceptive signaling originating from chemotherapy-damaged peripheral nerve fibers.
Neurochemical Modulation	Suppression of pro-inflammatory cytokine cascades and regulation of voltage-gated sodium (Na^+^) and calcium (Ca^2+^) channels.	Attenuates peripheral nerve hyperexcitability and neuropathic pain transmission.
Endogenous Analgesic Systems	Triggered secretion of endogenous opioids, serotonin (5-HT), and noradrenaline (NE), stimulating descending inhibitory pain pathways.	Fortifies endogenous pain-suppression pathways and diminishes central pain perception.
Neuroimmune Modulation	Downregulation of neuroinflammatory cytokines and suppression of spinal glial cell (microglia/astrocyte) reactivity.	Mitigates ongoing neuroinflammation responsible for chronic, persistent neuropathic pain.
Autonomic Modulation	Upregulation of parasympathetic (vagal) tone alongside suppression of sympathetic hyperreactivity.	Restores autonomic equilibrium and supports systemic anti-inflammatory signaling.
Central Neuroplasticity	Reorganization and functional modulation of cerebral networks governing sensory-discriminative, affective, and cognitive aspects of pain processing.	Reshapes central pain processing, altering overall symptom perception and affective pain burden.

## Data Availability

No new data were created or analyzed in this study. Data sharing is not applicable to this article.
